# Current News

**Published:** 1896-02

**Authors:** 


### Current News.

### ODONTOGRAPHIC SOCIETY OF CHICAGO.

THE annual election of officers of the Odontographic Society of Chicago, held December 9, 1895, resulted as follows: President, Dr. C. E. Meerhoff; Vice-President, Dr. E. R. Carpenter; Secretary, Dr. H. H. Wilson; Treasurer, Dr. Edmund Noyes.

*Board of Directors.*—Dr. R. B. Fuller, Dr. C. E. Bentley, Dr. J. G. Reid.

*Board of Censors.*—Dr. A. B. Allen, Dr. H. A. Drake, Dr. G. W. Schwartz.

H. H. WILSON,
*Secretary.*

### TOPICS FOR DISCUSSION BY STATE AND LOCAL ORGANIZATIONS.

1. Should not the appointment of Dental Examining Boards be under the control of the State dental societies?

2. Should not the granting of certificates of qualification by examining boards to non-graduates be generally abolished?

3. To what extent is the washing of amalgam masses an important feature in the production of a gold filling?

4. What results are to be expected in replantation or transplantation as a means of treatment of chronic phagedenic pericementitis?

5. The committee earnestly recommend that the report of the Committee on Dental Nomenclature of the American Dental Association be fully studied and discussed.

LOUIS JACK,
J. N. CROUSE,
EDWARD C. KIRK,
*Committee on State and Local Organization.*

